# Allogeneic Lymphocyte Transfer in MHC-Identical Siblings and MHC-Identical Unrelated Mauritian Cynomolgus Macaques

**DOI:** 10.1371/journal.pone.0088670

**Published:** 2014-02-11

**Authors:** Edward T. Mee, Richard Stebbings, Joanna Hall, Elaine Giles, Neil Almond, Nicola J. Rose

**Affiliations:** 1 Division of Virology, National Institute for Biological Standards and Control, Medicines and Healthcare products Regulatory Agency, South Mimms, Hertfordshire, United Kingdom; 2 Division of Biotherapeutics, National Institute for Biological Standards and Control, Medicines and Healthcare products Regulatory Agency, South Mimms, Hertfordshire, United Kingdom; 3 Division of Biological Services, National Institute for Biological Standards and Control, Medicines and Healthcare products Regulatory Agency, South Mimms, Hertfordshire, United Kingdom; Commissariat a l'Energie Atomique(cea), France

## Abstract

The detailed study of immune effector mechanisms in primate models of infectious disease has been limited by the inability to adoptively transfer lymphocytes from vaccinated animals into naïve immunocompetent recipients. Recent advances in our understanding of the Major Histocompatibility Complex diversity of Mauritian cynomolgus macaques enabled the establishment of a breeding program to generate Major Histocompatibility Complex (MHC)-identical animals. The current study utilised this resource to achieve an improved model of adoptive transfer of lymphocytes in macaques. The effect of route of transfusion on persistence kinetics of adoptively transferred lymphocytes was evaluated in an autologous transfer system. Results indicated that peripheral persistence kinetics were comparable following infusion by different routes, and that cells were detectable at equivalent levels in lymphoid tissues six weeks post-infusion. In a pilot-scale experiment, the persistence of adoptively transferred lymphocytes was compared in MHC-identical siblings and MHC-identical unrelated recipients. Lymphocytes transferred intra-peritoneally were detectable in the periphery within one hour of transfer and circulated at detectable levels in the periphery and lymph nodes for 10 days. Donor lymphocytes were detectable at higher levels in MHC-identical siblings compared with unrelated animals, however the total time of persistence did not differ. These results demonstrate a further refinement of the lymphocyte adoptive transfer system in Mauritian cynomolgus macaques and provide a foundation for hitherto impractical experiments to investigate mechanisms of cellular immunity in primate models of infectious disease.

## Introduction

Primate models of infectious disease have enabled significant developments in our understanding of immunity against infection. In particular, infection of cynomolgus and rhesus macaques with Simian Immunodeficiency Virus (SIV) has enabled detailed modelling of Human Immunodeficiency Virus (HIV) pathogenesis [1]–[5], development and testing of antiviral drugs [6]–[9] and most notably the demonstration that sterilising immunity against immunodeficiency viruses can be achieved by inoculation of animals with live attenuated virus [10]–[14]. The latter provides hope that an effective vaccine can be developed against HIV. Though the live attenuated vaccine strategy cannot be applied in the clinic, definition of the immune effectors of the observed protection could permit replication of the effect using safer vaccine methods.

Studies of mechanisms of protection in murine models exploit the ability to transfer immune serum and lymphocytes, or lymphocyte fractions, from vaccinated or convalescent animals to naïve recipients prior to challenge. To unravel the protection conferred by live attenuated SIV, serum transfer has been performed in the macaque/SIV model, with some studies suggesting that protection can be transferred in some situations [15], [16], whereas in others no effect was evident [17]. Until recently, the outbred nature of macaques has precluded the transfer of lymphocytes, preventing the investigation of whether acquired cellular immunity is a component of the robust protection induced by live attenuated SIV. The identification of a population of genetically restricted cynomolgus macaques on the island of Mauritius (hereafter referred to as Mauritian cynomolgus macaques, MCM) presented the exciting possibility of performing lymphocyte adoptive transfer in a primate model of infectious disease. Specifically, the Major Histocompatibility Complex (MHC) repertoire of wild and captive-bred MCM is represented by just eight haplotypes, six of which comprise >99% of the total MHC diversity [18], [19]. The identification of MHC-identical MCM is thus simpler than in the more outbred primate populations generally used in biomedical research.

Successful transfer of lymphocytes between MHC-identical MCM was demonstrated by Greene et al., with the donor cells detectable for up to 14 days in the recipient animal [20]. We sought to improve the methodology in three ways. Firstly; while the identification of small numbers of MHC-identical MCM is relatively straightforward in captive populations, the long generation time of macaques relative to other laboratory animals means that suitable experimental animals would be rapidly depleted from the population. To ensure continued supply of suitable genotyped and age-matched animals, we established several selective breeding groups of MCM [18], which have been productive for four years at the time of writing. Secondly, we investigated whether the route of infusion of transferred cells affected persistence time or kinetics, as has been reported in a rhesus macaque adoptive transfer model [21]. Finally, we reasoned that adoptively transferred lymphocytes would persist for longer in MHC-identical siblings than they would in MHC-identical unrelated animals, due to increased sharing of Minor Histocompatibility Antigens, Killer Immunoglobublin-like Receptors and other polymorphic proteins. Here we present data demonstrating that the route of infusion does not affect persistence in this model system, and that transferred cells appear and persist at higher levels in the periphery, though not for a longer time, in MHC-identical siblings compared with MHC-identical unrelated animals.

## Results

### Equivalent persistence kinetics of adoptively transferred lymphocytes administered intra-venously and intra-peritoneally

**Figure 1:**
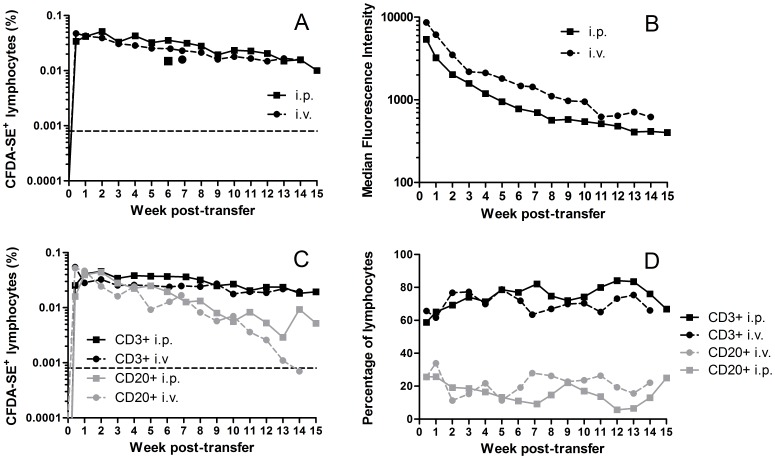
Persistence of adoptively transferred autologous lymphocytes. ***A*** Percentage of CFDA-SE+ lymphocytes in peripheral blood. Individual symbols represent lymph node biopsy samples; ***B*** Median fluorescence intensity of CFDA-SE+ cells; ***C*** Percentage of lymphocyte sub-populations positive for CFDA-SE; ***D*** Percentage of CD3+ and CD20+ cells in lymphocyte fraction.

In order to investigate whether lymphocyte persistence was affected by the route of infusion of the donor cells, we performed sequential autologous lymphocyte transfers in a single animal. In the first transfer, PBMC were isolated, stained with carboxyfluorescein diacetate succinimidyl ester (CFDA-SE) and 3×10^7^ cells reinfused intra-peritoneally in a volume of 5 ml saline containing 2% (v/v) autologous serum. The second transfer was administered intra-venously following decay of CFDA-SE staining to near-background levels. Following both i.p. and i.v. transfer, labelled cells were detectable in peripheral blood after three days. Lymphocyte persistence was equivalent by both methods, with cells comprising a relatively stable proportion (0.01–0.05%) of total lymphocytes over a period of 14–15 weeks (Fig. 1A). The decay in fluorescence intensity of the labelled cells did not suggest significant replication of the donor cells, indicating that the *ex vivo* manipulation had not unduly altered the cell phenotype (Fig. 1B). The proportion of labelled CD20+ lymphocytes in the periphery declined more rapidly than that of CD3+ cells (Fig. 1C), though no notable alterations in total CD3+ or CD20+ lymphocyte populations were observed (Fig. 1D). In addition to blood samples, a peripheral lymph node (PLN) biopsy was taken at six (i.p.) or seven (i.v.) weeks post-infusion. In both cases, CFDA-SE-labelled cells were detectable in the lymph node biopsy at levels comparable with those identified in the peripheral samples. Together, these results did not indicate any significant difference in the persistence of transferred cells according to the route of infusion. Due to the relative ease of i.p. compared with i.v. infusion, this route was selected for subsequent allogeneic lymphocyte transfer.

### Allogeneic lymphocytes circulate at higher levels in MHC-identical related compared with MHC-identical unrelated animals

**Figure 2:**
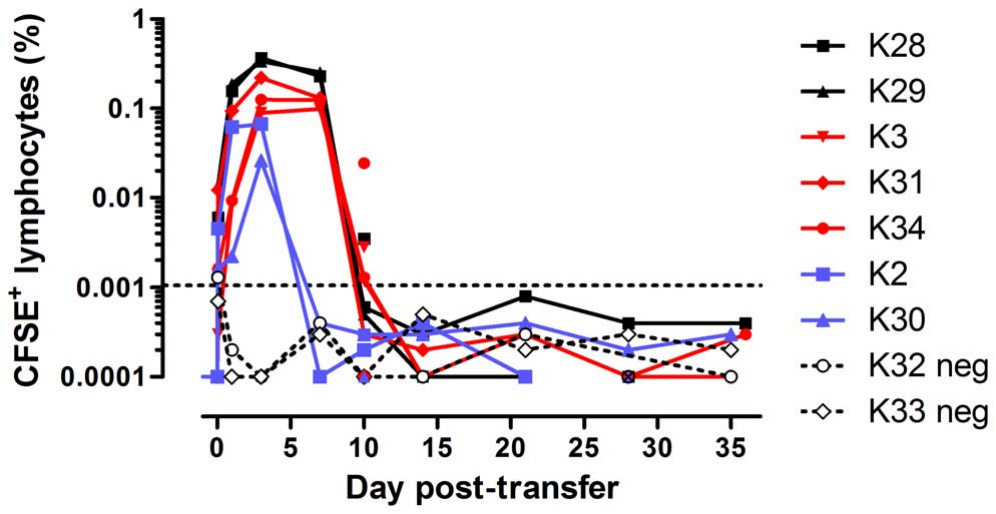
Persistence of adoptively transferred allogeneic lymphocytes in recipient animals. Solid lines represent peripheral blood samples; individual symbols represent lymph node biopsy samples. Dashed line indicates threshold for detection of CFDA-SE+ cells, calculated based on the mean of all negative samples plus two standard deviations. Animals are colour-coded by experimental group: *black* MHC-identical siblings; *red* MHC-identical unrelated; *blue* MHC-mismatch; *neg* negative control untreated animals.

Following termination of the donor animal, lymphocytes were isolated from blood, spleen and lymph nodes and labelled with CFDA-SE. A total of 4×10^8^ pooled lymphocytes was infused i.p. in a volume of 20 ml saline containing 2% (v/v) autologous (to the recipient animal) serum. The pool comprised 7% PBMC, 23% splenocytes and 70% mesenteric lymph node (MLN)- derived lymphocytes and contained 80% CD3+ and 18% CD20+ lymphocytes. No adverse reactions were observed following transfer. Lymphocyte persistence was monitored as for the autologous transfer. Donor cells were observed in the periphery within one hour of transfer in most animals, with levels exceeding 0.1% of total circulating lymphocytes at 24 hours (Fig. 2). Maximal levels (0.026–0.368%) were achieved in most animals by day 3, after which levels declined gradually at day 7 and then sharply at day 10. By day 14 post-infusion, CFDA-SE-labelled cells were below the limit of detection in all animals. As expected, transferred cells were eliminated most rapidly from MHC-mismatched animals. While the persistence time was equivalent in both MHC-identical groups, the proportion of transferred cells was 2–4-fold greater in both MHC-identical siblings than in the MHC-identical unrelated groups. The sibling animals were smaller (average 2 kg) than the unrelated animals (average 2.8 kg) at the time of infusion (Table 1), thus the donor cells would represent a larger proportion of total lymphocytes in these animals. The differences in weight did not, however, appear sufficient to explain the observed effect – the differences in weights were 1.2- to 1.8-fold while the differences in proportion of CFDA-SE-labelled cells at day 3 were 1.7- to 4-fold. A peripheral lymph node biopsy was taken from each animal 10 days post-transfer. CFDA-SE-labelled cells were detected in three of five MHC-matched recipients, confirming that transferred cells had migrated to lymphoid tissues (Fig. 2). Donor cells represented a greater proportion of lymph-node lymphocytes at day 10 than the corresponding fraction of peripheral lymphocytes at the same time point. A second lymph node biopsy was taken from five animals at day 28 post-transfer. There was no evidence of donor cells in these samples.

**Table 1:** Experimental groupings and MHC genotype of cynomolgus macaques.

Experimental group	ID	MHC genotype	Sex	Age^a^	Weight (kg)^a^	Notes
Donor animal	K1	M1, M1	Male	2.8	3.67	
MHC-identical siblings	K28	M1, M1	Female	1.9	2.61	Full sibling of K1
	K29	M1, M1	Female	2.1	2.57	Half sibling of K1. Sacrificed day 22
MHC-identical unrelated	K3	M1, M1	Male	3.1	4.64	Sacrificed day 22
	K31	M1, M1	Female	3.2	3.19	
	K34	M1, M1	Female	2.1	3.27	
MHC-mismatched	K2	M6/2 rec, M6/3 rec	Male	2.5	2.67	Sacrificed Day 22
	K30	M5, M6/2 rec	Female	3.0	4.18	
Untreated controls	K32	M1, M3	Female	3.6	3.19	
	K33	M1, M3	Female	3.1	5.30	

*rec*, recombinant MHC haplotype; a, reported ages and weights were on the day of allogeneic lymphocyte transfer.

### Limited evidence of persisting transferred lymphocytes in lymphoid tissues at 22 days post-transfer

**Figure 3:**
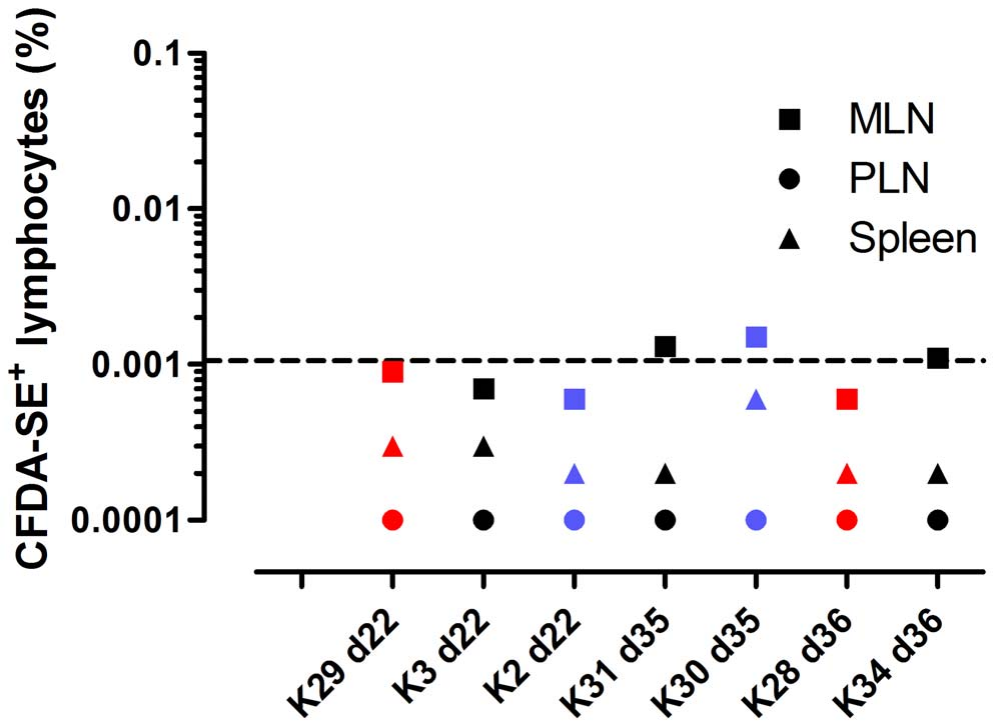
Detection of adoptively transferred allogeneic lymphocytes in lymphoid tissues of recipient animals at days 22, 35 and 36 post-transfer. Dashed line indicates threshold for detection of CFDA-SE+ cells. Animals are colour-coded by experimental group: *black* MHC-identical siblings; *red* MHC-identical unrelated; *blue* MHC-mismatched.

One animal from each experimental group was sacrificed at day 22 and spleen, mesenteric lymph nodes (MLN) and peripheral lymph nodes (PLN) were sampled. CFDA-SE-labelled cells were undetectable in all samples (Fig. 3). The remaining four animals were sacrificed at day 35-36 post-transfer and samples taken as above. Low level CFDA-SE+ populations were observed in the MLN of two animals – however, the absolute number of events was low (10–13 of one million events) and the signal was close to the threshold of detection. Together these results indicate that persistence of cells in the tissues was either low level or undetectable regardless of experimental group.

## Discussion

Following demonstration of the feasibility of adoptive transfer of lymphocytes in MHC-matched but unrelated MCM [20], we reasoned that the model could be further refined by the use of related animals, and by determining whether alternative routes of infusion (apart from intravenous transfer) resulted in improved persistence of the transferred cells. Here, we have presented data demonstrating no difference in the kinetics of lymphocyte persistence irrespective of whether cells were transferred intra-peritoneally or intravenously. Second, we observed that whilst cells persisted at higher levels, the duration of persistence was no greater in MHC-matched related compared to unrelated animals.

Following autologous transfer of PBMC, labelled cells were detectable at a relatively stable frequency in the periphery for up to 15 weeks. The more rapid decline in the frequency of labelled CD20+ relative to CD3+ lymphocytes may be indicative of more rapid turnover of CD20+ cells or their migration to tissues such as spleen and lymph node, though the investigation of this possibility was beyond the scope of the current experiment. The gradual decline in the fluorescence intensity of the labelled cells suggested that limited, if any, proliferation had resulted from the *ex vivo* manipulation of the cells. Notably, no difference in the kinetics of persistence was observed between the two routes of administration. This result contrasts with the finding of Bolton et al. [21] that cells transferred i.p. persist at initially lower but more stable levels than those transferred i.v.. The discrepancy may be due to the use of different cell populations – the activated and *ex vivo* cultured T lymphocytes used in that study were likely morphologically and phenotypically distinct from the resting total lymphocyte populations transferred in our study and may therefore have preferentially trafficked to, or become trapped in, tissues. The cell populations likely to be of most direct interest in an adoptive transfer model for defining correlates of immunity are total (or fractionated) uncultured lymphocytes from an immune animal. Our data therefore provide important information on the route of administration. One issue that could not be fully addressed by autologous transfer was the effect of different routes of infusion on trafficking of cells to various lymphoid tissues. Data from lymph node biopsies suggested no difference in that tissue. However, more invasive biopsies to investigate MLN and gut-associated lymphoid tissue (GALT) could not be performed in this study. Nevertheless, given the available data, logistical ease and larger volume that can be administered intra-peritoneally, this route would seem preferable for future adoptive transfer studies.

Following transfer of allogeneic lymphocytes to recipient animals, cells were detectable in the periphery within one hour in most animals, and by 24 hours in all except negative control animals, suggesting that rapid trafficking of cells from the peritoneum had occurred. Cells reached peak levels in the periphery after three days, comprising up to 0.37% of the total lymphocyte fraction, but thereafter declined rapidly and were undetectable by 10 days post-transfer in all but one animal. This result was consistent with the 9–17 day persistence of bulk lymphocytes reported by Greene et al. [20], [22]. As expected, transferred cells were rapidly eliminated from MHC-mismatched recipients. Notably, whilst cells did not persist for longer in the periphery of MHC-matched sibling recipients, the levels were considerably higher than in MHC-matched unrelated animals. The low numbers of animals in this pilot scale experiment preclude statistical analysis; however this result may suggest that a greater number of cells survive during the initial phase of circulation. Crucially, the successful transfer of a sterilising immune response will depend on the suppression of pathogen replication during the first few days of exposure; hence there should be an advantage of using MHC-matched sibling animals for such studies. The most obvious explanation for this observation is that a greater degree of histocompatibility between donor and recipient cells allowed a greater number of cells to enter and circulate in the bloodstream, though the precise mechanism could not be addressed within the current study.

The reasons for the rapid disappearance of transferred cells from the periphery despite MHC compatibility remain unclear. Results from the autologous transfer suggested the *ex vivo* manipulation did not result in excessive proliferation (which would lead to rapid decay of CFDA-SE staining) and the initial decay in staining intensity of transferred cells, albeit based on only three time points, was comparable with that from the autologous transfer (data not shown). A second possibility for the rapid loss of circulating lymphocytes is that the cells preferentially trafficked to lymphoid tissues. The transferred cells comprised primarily MLN and splenic lymphocytes, likely expressing homing markers from their tissues of origin. Analysis of lymph node biopsies at day 10 and 28, and spleen samples at days 22 and 35 did not however, identify significant populations of labelled cells. It remains a possibility that the cells trafficked to these tissues and remained at low levels, but were undetectable by the staining methods employed in our study.

The final possibility is that, despite MHC matching, the transferred cells were recognised as foreign and destroyed by the recipient immune system. This possibility could be formally addressed by transferring cells into immunosuppressed macaques; if non-self recognition were the major reason for elimination of transferred cells the donor cells would be predicted to persist for longer in immunosuppressed recipients. Aside from MHC, Killer cell Immunoglobulin-like Receptors (KIR) and Minor Histocompatibility Antigens are the most likely antigens to be recognised as foreign. While matching for these antigens could in theory be achieved, the numbers of available animals would rapidly diminish with each additional matched factor. Our approach of breeding all MHC-matched siblings from a single sire will have at least partially addressed this issue as all the offspring inherited only one of two paternal KIR and Minor Histocompatibility Antigen haplotypes. As successive generations of animals are bred in this matrilineal colony, the diversity of these loci should constrict considerably and the inclusion of a male homozygous for KIR (for which only eight haplotypes are present at appreciable frequencies in MCM [23]) and Minor Histocompatibilty Antigen haplotypes would further control for these factors.

Our study shared commonalities with that recently reported by Greene et al. [22]. Discrepancies in results were apparent though these can largely be explained by differences in experimental design. Of note, that study reported circulating frequencies of transferred cells as high as 27% after five minutes and 2.67% after seven days. The peak level of circulating donor lymphocytes in our study was 0.37% after three days; however the composition of the transferred cells differed and we transferred approximately 4–7-fold fewer cells than Greene et al. The kinetics of persistence varied markedly, with Greene et al. reporting high initial levels of circulating donor cells followed by rapid and significant decline within 48 hours, by contrast with the initial low levels (at one hour) followed by rise and then fall observed in our study. This likely reflects both the gradual migration of cells from the peritoneum into the circulation (compared with the immediate introduction of cells intra-venously) and differences in composition of the transferred cells between the two studies. Another difference was that Greene and colleagues transfused cells intra-venously whilst we selected the intra-peritoneal route; our autologous transfer results would, however, suggest that this difference would only affect persistence kinetics in the periphery in the first few hours following transfusion. Our experimental design focussed on addressing the issue of lymphocyte persistence and therefore did not include a live attenuated vaccination/wild-type challenge. This is, however, the most immediate application of an adoptive lymphocyte transfer model that has now been demonstrated to be viable in three separate reports. In addition to further refinements to the host side of the model, e.g. by selectively breeding animals from animals sharing KIR and/or Minor Histocompatibility Antigens as well as MHC, the use of a conditionally live attenuated SIV, dependent upon the presence of doxycycline to replicate [24], [25] and capable of infecting macaques [26] provides a means of preventing the replication of the vaccine virus in the recipient which may confound the interpretation of previously reported studies. These tools would advance our understanding of this intriguing model of potent vaccine-mediated protection against SIV and inform AIDS vaccine development.

We have demonstrated higher levels of circulating donor lymphocytes in siblings compared with unrelated animals receiving lymphocyte transfers from an MHC-matched donor. Though the persistence was not extended, the higher levels of cells suggest a more complete and potentially functionally competent population of lymphocytes. Any study of sterilising immunity in SIV models will centre on immunity in the first hours and days following challenge, thus the higher levels of circulating cells may be crucial in allowing a mechanism of protection to be dissected. There remain practical challenges in the breeding of fully histocompatible cynomolgus macaques, but our results support such a programme as a worthwhile objective to enable the detailed immunological investigations necessary for a clear definition of the immune correlates of protection against SIV.

## Methods

### Animals and ethics statement

Juvenile, weaned, cynomolgus macaques of defined genotype were selected for the experiment. Predicted MHC haplotypes (specifically homozygosity for haplotype M1 based on parental information [18]) were confirmed by microsatellite-based genotyping according to the method of Wiseman et al [19]. Animals were housed in two same-sex groups of 3–4 animals in cages which conform to EU guidelines on dimensions, with daily feeding and access to water *ad libitum*. The environmental temperature was 15–24°C, appropriate for macaques and rooms were subject to a 12 hour day/night cycle of lighting. A variety of environmental enrichment was provided, including toys encouraging foraging for food, swings and perching stations at different heights. Bedding material was wood shavings. Animals were acclimatised to their environment and deemed to be healthy by the named Veterinary Surgeon prior to inclusion on the study. This work was performed under a licence granted by the Secretary of State for the Home Office according to the Animals (Scientific Procedures) Act 1986. As part of the process of application, detailed plans of the procedural work are reviewed by the institution's Ethical Review Process as well as external scrutiny for animal welfare, before the licence is granted. Given the limited availability of suitable animals, age, weight and sex matching were not possible and not deemed necessary for the study success – characteristics of each animal are detailed in Table 1.

### Experimental procedures and sampling

All procedures were performed under sedation with ketamine hydrochloride. Prior to the allogeneic lymphocyte transfer, donor animal K1 was immunised three times with a pool of five SIVmac239 peptides as part of a parallel study. Blood samples for monitoring were taken into EDTA. Blood samples for transfer were taken into heparin. Animals were humanely killed by overdose of anaesthetic at the end of the study period.

### Autologous transfer

PBMC were isolated from 15 ml whole blood using Ficoll Paque Plus (GE Healthcare, Hatfield, UK), residual erythrocyte contamination was removed using ammonium chloride potassium (ACK) lysis buffer (Life Technologies, Paisley, UK) and cells washed in PBS. Cells were labelled with 5µM CFDA-SE (Life Technologies) for exactly 10 minutes at 37°C and the reaction was stopped by the addition of PBS containing 10% autologous serum that had not been heat-treated. Cells were washed with PBS and resuspended in 0.9% sterile sodium chloride containing 2% autologous serum. For the intra-peritoneal transfer, 3.12×10^7^ cells (>99% viability) were infused in a total volume of 5 ml. For the intra-venous transfer, 2.65×10^7^ cells were infused in a total volume of 5 ml. Total time from blood draw to infusion was approximately three hours.

### Allogeneic transfer

Animal K1 was sacrificed by overdose of ketamine hydrochloride. Blood was taken into heparin and spleen and mesenteric lymph nodes into RPMI 1640 (Life Technologies). Spleen and lymph nodes were crushed using a syringe plunger and filtered through a 70 µm filter (Life Technologies). PBMC and splenocytes were isolated using Ficoll Paque Plus, treated with ACK lysis buffer and resuspended in PBS. MLN were used directly following filtration. Cells were labelled as above and the reaction was quenched with PBS containing 10% serum from the donor animal. Cells were pooled and resuspended in seven lots of 20 ml sterile 0.9% sodium chloride, each containing 2% serum from the relevant recipient animal. Cells were infused intra-peritoneally approximately 5.5 hours following termination of the donor. Each infusion contained 4.8×10^8^ cells, of which 4.1×10^8^ cells were viable based on ethidium bromide/acridine orange staining. All animals were immunised with a pool of SIV peptides 14 days post-transfer as part of a parallel study.

### Detection and immunophenotying of CFDA-SE-labelled donor cells by flow cytometry

For detection of total CFDA-SE+ cells, 500 µl whole blood was added to a 5 ml polystyrene tube (BD Bioscience, Oxford, UK) with 4.5 ml ACK lysis buffer and incubated for 10-15 minutes at room temperature. Cells were pelleted by centrifugation at 400×g, washed once with ACK lysis buffer, once with Cell Wash (BD Bioscience) containing 1% foetal bovine serum and resuspended in 2.5 ml 2% formaldehyde in 0.5× PBS. Immunophenotyping was perfomed as above, with the addition of antibodies specific for CD3 (APC, clone 10D12, Miltenyi Biotec, Surrey, UK) and CD20 (PE, clone H299, Beckman Coulter, High Wycombe, UK) to the whole blood followed by incubation for 20 minutes at room temperature. One million events in the lymphocyte gate (as determined by forward scatter and side scatter characteristics) were collected for each sample on a FACS CantoII flow cytometer (BD Bioscience), with spectral compensation applied using single stained controls. Data were analysed using FACS Diva v6 (BD Bioscience) and data were plotted using Prism v5 (GraphPad software, La Jolla, CA, USA).

## Supporting Information

Checklist S1 **The ARRIVE Guidelines Checklist.**
(DOC)
